# Quantification and dosimetry of small volumes including associated uncertainty estimation

**DOI:** 10.1186/s40658-022-00512-9

**Published:** 2022-12-13

**Authors:** Lily Carnegie-Peake, Jan Taprogge, Iain Murray, Glenn D. Flux, Jonathan Gear

**Affiliations:** 1 Joint Department of Physics, Royal Marsden NHSFT, Downs Road, Sutton, SM2 5PT UK; 2 The Institute of Cancer Research, 15 Cotswold Road, Sutton, SM2 5NG UK

**Keywords:** Quantification, Dosimetry, I-131, Uncertainty estimation

## Abstract

**Background:**

Accurate quantification of radioactivity in a source of interest relies on accurate registration between SPECT and anatomical images, and appropriate correction of partial volume effects (PVEs). For small volumes, exact registration between the two imaging modalities and recovery factors used to correct for PVE are unreliable. There is currently no guidance relating to quantification or the associated uncertainty estimation for small volumes.

**Material and methods:**

A method for quantification of small sources of interest is proposed, which uses multiple oversized volumes of interest. The method was applied to three Na[^131^I]I activity distributions where a Na[^131^I]I capsule was situated within a cylindrical phantom containing either zero background, uniform background or non-uniform background and to a scenario with small lesions placed in an anthropomorphic phantom. The Na[^131^I]I capsule and lesions were quantified using the proposed method and compared with measurements made using two alternative quantification methods. The proposed method was also applied to assess the absorbed dose delivered to a bone metastasis following [^131^I]mIBG therapy for neuroblastoma including the associated uncertainty estimation.

**Results:**

The method is accurate across a range of activities and in varied radioactivity distributions. Median percentage errors using the proposed method in no background, uniform backgrounds and non-uniform backgrounds were − 0.4%, − 0.3% and 1.7% with median associated uncertainties of 1.4%, 1.4% and 1.6%, respectively. The technique is more accurate and robust when compared to currently available alternative methods.

**Conclusions:**

The proposed method provides a reliable and accurate method for quantification of sources of interest, which are less than three times the spatial resolution of the imaging system. The method may be of use in absorbed dose calculation in cases of bone metastasis, lung metastasis or thyroid remnants.

**Supplementary Information:**

The online version contains supplementary material available at 10.1186/s40658-022-00512-9.

## Background

Accurate quantification of single-photon emission computed tomography (SPECT) imaging is essential for absorbed dose calculations in molecular radiotherapy (MRT) and offers the potential to provide additional information for diagnostic nuclear medicine studies [1–3]. The uptake of activity within an organ or lesion requires delineation of a volume of interest (VOI) on the SPECT image, taking into account either the anatomical or functional boundaries. Anatomical information provided by computed tomography (CT) aids in this approach [4, 5]. However, the transfer of anatomical outlines onto the SPECT image relies on exact registration between SPECT and CT, which may be hindered by patient motion. Mis-registration between these two modalities becomes more prominent for small volumes, such as bone and lung lesions, or thyroid remnants.

The finite resolution of the SPECT system also presents difficulties with image quantification due to the partial volume effect (PVE) [6]. Several methods have been proposed to overcome this problem, including the application of a recovery coefficient to correct for the observed “spill out” of activity from the anatomical volume [5, 7]. Recovery curves can be generated by plotting measured recovery coefficients for different volumes and fitting a function to the data [8]. These usually demonstrate a steep gradient at smaller volumes [9–11]. An error in the volume estimate at small volumes therefore leads to a large uncertainty in the applied recovery coefficient and accuracy of the final quantification [12]. Object shape may also influence quantification accuracy as recovery coefficients are often determined for simplified shapes such as spheres or cylinders [13]. Similarly, results may also be affected by septal penetration, especially for high-energy gamma emitters such as ^131^I [12].

Quantification using an oversized VOI that encompasses all counts originating from the source of interest [14, 15] is potentially a more appropriate approach. However, a correction that accounts for the inclusion of neighbouring counts in the VOI may then be necessary. Typically, this correction is performed using a second local VOI [14]. The size and position of the oversized VOI and that used for correction of neighbouring counts can affect the measurement. This is particularly true if the VOI is close to a secondary site of increased radiopharmaceutical uptake.

There is currently no guidance regarding uncertainty estimation of quantification of small sources of interest. European Association of Nuclear Medicine (EANM) guidelines for estimating uncertainties were demonstrated for lesions greater than 3 times the spatial resolution of the imaging system [16], and Finocchiaro et al. [17] demonstrated that the uncertainty estimates of dosimetric parameters for small volumes can escalate to such an extent that the uncertainty exceeds the value of the associated parameter. Linearisation using a first-order partial derivative as described in the EANM guideline is then no longer valid [18].

An alternative quantification and uncertainty estimation method is proposed. The approach uses multiple concentric outlines to determine the activity of a source. The technique enables accurate correction by modelling the contribution of surrounding activity to the counts contained within the concentric VOIs and estimates the associated uncertainty. Furthermore, as all activity within the site of interest is included in the VOI there is no need to apply a recovery coefficient, which is otherwise an additional source of uncertainty in the dose calculation [16].

The approach is validated by applying the technique to known activity distributions in phantoms designed to represent increasingly complex scenarios. Application to a clinical case study is presented that assesses the absorbed dose delivered to a bone metastasis following [^131^I]mIBG therapy for neuroblastoma.

## Theory

An oversized VOI on the SPECT image is delineated that encompasses all counts originating from the source of interest. A minimum of two additional concentric VOIs of increasing volume are also delineated around the initial oversized VOI. The total counts measured within each VOI are a summation of those originating from the source of interest as well as radioactivity in surrounding tissue. As the VOI size increases, the contribution from the surrounding radioactivity is assumed to increase linearly. Under the assumption that all VOIs contain 100% of counts originating from the source of interest, the y-intercept of a linear plot of VOI counts,$C_{i}$, versus VOI volume,$v_{i}$, represents events originating solely from the source of interest.

**Fig. 1:**
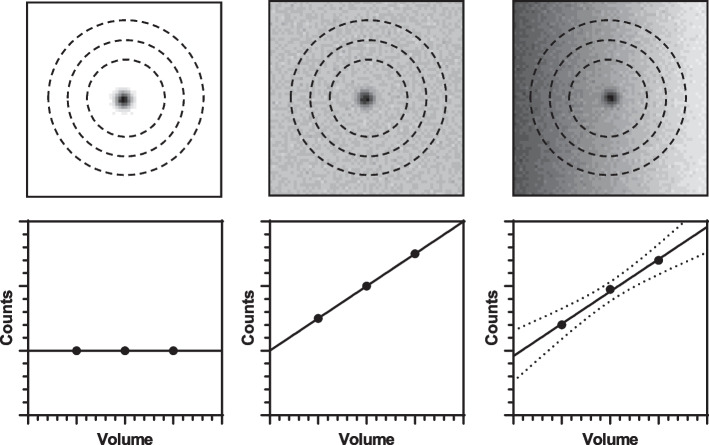
Illustration of linear extrapolation to y-intercept of cold, uniform, and anisotropic background using concentric oversized VOIs

Figure 1 presents an illustration of this technique, for three different scenarios. In the first, there is no activity surrounding the source, and therefore, the counts recorded in each VOI are equal to the intercept, $C_{v = 0}$. In the second scenario, uniform neighbouring activity surrounds the source, and the counts within each VOI decrease linearly. In the third case, the background is non-uniform and the counts do not perfectly follow a straight line. A linear fit to these data results in an uncertainty in the intercept value, which is used as the uncertainty in source counts.

The counts associated with the source are defined as the intercept of the fitted function,

1 $$C_{v = 0} = \frac{{\left( {n\sum v_{i}^{2} } \right)\left( {n\sum {\text{C}}_{i} } \right) - \left( {n\sum v_{i} } \right)\left( {n\sum v_{i} C_{i} } \right)}}{{\left( {n\sum v_{i}^{2} } \right) - \left( {\sum v_{i} } \right)^{2} }},$$

where $C_{i}$ is the number of counts within the VOI of volume, $v_{i}$, and $n$ is the number of VOIs. The uncertainty in the intercept is given by:

2 $$u^{2} \left( {C_{v = 0} } \right) = \frac{{\sum \left( {C_{i} - av_{i} - C_{v = 0} } \right)^{2} }}{n - 2}\frac{{\sum v_{i}^{2} }}{{\left( {n\sum v_{i}^{2} } \right) - \left( {\sum v_{i} } \right)^{2} }}$$

where $a$ is the gradient of the slope, equivalent to the average background count concentration. The expression to determine the activity $A$, from the measured counts, is as follows:

3 $$A = \frac{C}{QR}$$

where $Q$ is a quantification or calibration factor that describes the system sensitivity (i.e. the expected counts per unit activity) and $R$ is the recovery coefficient. In the case of an oversized VOI, all counts have been included and the recovery term is unity.

Using the law of propagation of uncertainty (LPU), the uncertainty associated with the estimate of activity,$u\left( A \right)$, is then expressed as,

4 $$\left[ {\frac{u\left( A \right)}{A}} \right]^{2} = \left[ {\frac{u\left( Q \right)}{Q}} \right]^{2} + \left[ {\frac{u\left( C \right)}{C}} \right]^{2} .$$

The proagation of uncertainty in activity to that associted with absorbed dose is described further in Additional file 1.

## Methods

### Phantom acquisitions

A 2.8 MBq sodium iodide (Na[^131^I]I) capsule was measured in a Fidelis (Southern-Scientific, Henfield, UK) secondary standard dose calibrator. The standard uncertainty associated with the repeated measurements was combined with the quoted uncertainty in the calibration factor to ascertain a total uncertainty of capsule activity. To test the proposed approach, SPECT/CT acquisitions were performed using the Na[^131^I]I capsule situated within a plastic sample tube and placed within the centre of a water-filled cylindrical phantom (20 cm diameter). Different acquisitions were performed representing the three distribution scenarios described in Fig. 1.

An additional acquisition was performed using a modified anthropomorphic phantom with four point sources representing small lesions placed at various locations within the phantom.

In each case, the proposed method for estimating source activity was applied and the results compared to the known activity. Furthermore, results were compared to results using an oversized VOI with a local background VOI (“Local VOI method”) as well as the recovery coefficient (RC) method. The proposed approach was then used to calculate the absorbed dose delivered to a bone metastasis for a paediatric patient who received [^131^I]mIBG radionuclide therapy.

Gamma camera acquisitions were performed on a Siemens Symbia Intevo SPECT/CT system equipped with high-energy general-purpose collimators (HEGP). Seventy-two projections were acquired over 360 degrees for 60 s each. Projections were acquired on a 256 × 256 matrix but rescaled to 128 × 128 during reconstruction. Images were reconstructed using Hermes Hybrid-Recon Oncology 3.0.0 (Hermes Medical Solutions, Stockholm, Sweden). A 3D OSEM reconstruction algorithm was employed (5 iterations, 8 subsets) incorporating CT-based attenuation correction and Monte Carlo scatter correction. No resolution recovery was employed as this resulted in Gibbs-like artefacts around the point sources in most scenarios.

An oversized spherical VOI was placed within the reconstructed image, centred on the capsule, including all visible counts. The total count rate within the VOI, $C_{{{\text{VOI}}}}$, and the activity of the capsule at the midpoint of the scan, $A_{{{\text{capsule}}}}$, were used to determine a sensitivity factor, using:

5 $$Q = \frac{{C_{{{\text{VOI}}}} }}{{ A_{{{\text{capsule}}}} }}$$

#### Phantom configurations

##### No background: cylindrical phantom

Multiple validation acquisitions using the phantom and acquisition protocol described above were acquired. The capsule activity at the times of acquisition ranged between 3.3 and 33.3 MBq. The absolute activity in the capsule at each time point was determined using each of the three methods described. Uncertainty in activity was determined for both the proposed and RC methods using the schema described here and that described in the EANM guidelines [16], respectively. The absolute errors and uncertainty on the measurements were then plotted as a function of activity.

##### Uniform background: cylindrical phantom

To test the validity of the proposed approach in the presence of background activity, the capsule was removed from the phantom and a solution containing 46 kBq/ml of Na[^131^I]I used to fill the background compartment. The phantom, couch and detector positions were consistent with the capsule acquisitions. To simulate different source to background ratios, Poisson resampling [19] was performed on the projection data of the background phantom acquisition data. These data were then combined with one of the previously acquired no background capsule data prior to reconstruction.

The composite images were created for the highest (33.3 MBq) capsule activity with multiple background activity concentrations. The measured activity with associated uncertainties were then compared to the known capsule activity.

##### Non-uniform background: cylindrical phantom

Non-uniform backgrounds were created using non-uniform Poisson resampling to introduce a count gradient along the z-axis of the projection data acquired using a uniformly filled phantom. Four such non-uniform background datasets of differing count gradients were generated. These data were combined with one of the previously acquired no background capsule data (capsule activity = 33.3 MBq) prior to reconstruction. The local VOI method was performed for two different VOI positions as indicated in Fig. 7. The measured activity with associated uncertainties was then compared to the known capsule activity.

##### Anthropomorphic phantom

To test the methodology in a more clinically realistic geometry, a study using an anthropomorphic phantom was conducted. 3D-printed liver, spleen, right and left kidney inserts were manufactured to fit within the abdominal cavity of adapted Alderson Heart and Thorax Phantom (Radiology Support Devices, Inc., CA, USA). The background compartment and organs of the phantom were filled with Na[^131^I]I activities of 130.8, 77.2, 13.8, 17.0 and 13.4 MBq, respectively. Four point sources (approximately 0.12 ml in volume) were prepared with an activity concentration of 30 MBq/ml and attached at different locations within the phantom. The phantom was then scanned, and process as previously described.

### Quantification methods

#### Local VOI method

For comparative purposes, quantification was performed using a typical method of background correction. A VOI positioned locally to the capsule with total count rate, $C_{B}$, and volume, $V_{B}$, was used to determine activity using:

6 $$A = \frac{{C_{{\text{V}}} - \left( {\frac{{V_{{\text{V}}} }}{{V_{B} }} \cdot C_{B} } \right)}}{ QR}$$

where $C_{{\text{V}}}$ and $V_{{\text{V}}}$ are the total count rate and volume for a VOI which encompasses all counts originating from the source ($R = 1$).

#### The recovery coefficient method

The RC method for quantification was also used as a comparator. A recovery curve was determined for the system. Six spherical inserts with diameters ranging from 1.0 to 6.5 cm were filled with a solution containing 0.3 MBq/ml of Na[^131^I]I. SPECT/CT acquisitions were performed on the same system using identical scanning parameters to that described previously. Images were also reconstructed in an identical manner.

Spherical VOIs matching the physical insert size were delineated on the image and the total counts $C_{i}$ within each VOI corresponding to insert $i$ were recorded. A recovery factor for each insert was then determined using

7 $$R_{i} = \frac{{C_{i} Q}}{{A_{i} }},$$

where $A_{i}$ is the known activity in insert $i$. A two-parameter logistic function was fitted to the data with respect to volume $v$, namely [16]:

8 $$R\left( v \right) = 1 - \frac{1.}{{1 + \left( {\frac{v}{{b_{1} }}} \right)^{{b_{2} }} .}}$$

The counts measured within an anatomical VOI delineated on the CT image and transferred to the SPECT image were used to calculate activity in conjunction with Eqs. (3) and (8).

#### Clinical case study

The method was finally demonstrated on a relevant clinical dosimetry case, for a paediatric neuroblastoma patient undergoing [^131^I]mIBG radionuclide therapy. A single bone metastasis with visible uptake in the left pelvic bone was selected for dosimetry. SPECT/CT imaging was performed at 43, 115 and 167 h post-injection using the same acquisitions and processing methodologies previously described.

The bone metastasis was manually delineated by a trained nuclear medicine radiologist using data from both the [^131^I]mIBG SPECT/CT investigation and a previously acquired [^123^I]mIBG SPECT/CT. Activity at each time point was quantified using the proposed and RC methods and time activity curves fitted to the data. Time-integrated activity was determined by taking the integral of a single exponential function fitted to the data. Uncertainty analysis on the measurements followed the methods described above and that within the EANM guidance document. Dose factors were selected from a look-up of OLINDA spherical dose factors based on the volume of the delineated SPECT VOI. Absorbed dose was calculated using the general MIRD equation assuming negligible contribution to dose from sources of activity outside of the lesion.

## Results

System sensitivity for Na[^131^I]I measured on the Siemens Symbia Intevo for the acquisition and reconstruction procedure described was determined to be 28.9 ± 0.4 cps/MBq.

### Recovery curve characterisation

**Fig. 2:**
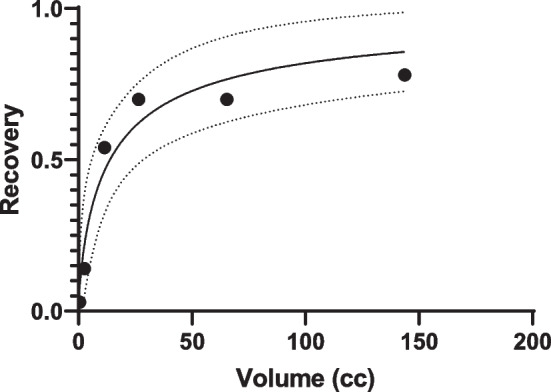
Recovery curve with 95% confidence intervals (dashed line) determined by an empirical fit to the spherical insert data

Recovery data generated from the phantom measurements are shown in Fig. 2. The empirical fit to the data is shown with uncertainty represented by a 95% confidence interval. The volume of the iodine capsule estimated from CT was 0.33 ± 0.17 cc which, using Fig. 2, corresponds to a required recovery coefficient of 0.05 ± 0.04.

### No background

**Fig. 3:**
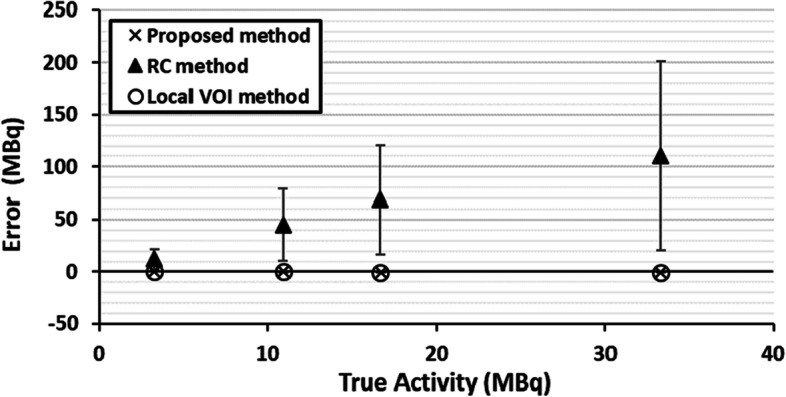
Absolute errors and uncertainties for the proposed method, RC method and local background VOI method for a capsule situated in the centre of a 20-cm-diameter cylindrical phantom filled with cold background (water) at four time points as the capsule decayed

Figure 3 summarises the quantification accuracy of the proposed, RC and local VOI methods with a source in cold background. The absolute errors are given for all methods, and uncertainties were calculated for the proposed and RC methods.

The proposed method resulted in an accurate quantification across the range of activities investigated (3.3–33.3 MBq), with all source activity estimates being within 0.5 MBq of the true activity. Median errors and uncertainties results are summarised in Table 1 for all three methods.

**Table 1:** Median errors and uncertainties in quantification using the proposed, RC and local VOI methods for three activity distributions

Phantom configuration	Method	Median error (MBq)	Median error (%)	Median uncertainty (%)
No background	Proposed	− 0.1 (− 0.41 to 0.07)	− 0.4 (− 1.3 to 0.6)	1.4 (1.38 to 1.41)
	RC	57 (12.5 to 110)	395 (333 to 412)	301 (271 to 313)
	Local VOI	− 0.1 (− 0.2 to 0)	− 0.3 (− 0.6 to 0)	N/A
Uniform background	Proposed	− 0.1 (− 0.2 to 0.1)	− 0.3 (− 0.6 to 0.2)	1.4 (1.36 to 2.03)
	RC	130 (106 to 142)	289 (215 to 326)	240 (195 to 264)
	Local VOI	1.4 (1.0 to 1.8)	4.2 (3.0 to 5.3)	N/A
Non-uniform background	Proposed	0.6 (0.4 to 1.1)	1.7 (1.1 to 3.4)	1.6 (1.5 to 1.7)
	RC	− 23 (− 23.4 to − 23.5)	− 70.3 (− 70.2 to − 70.4)	1.6 (1.5 to 1.7)
	Local VOI (position A)	6.7 (4.7 to 13.3)	20 (14 to 40)	N/A

Ranges are given in brackets

The local VOI method resulted in a degree of accuracy comparable to the proposed method. The recovery coefficient method produced the largest errors which increased with activity. For the RC method, uncertainty estimates were consistent with the observed error and increased with capsule activity.

### Uniform background

**Fig. 4:**
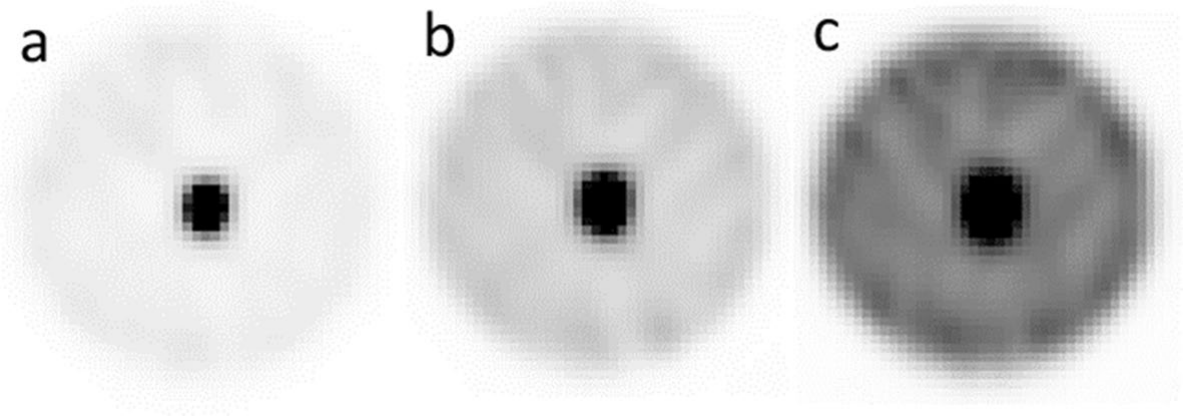
Capsule and background composite images. Uniform background generated by Poisson resampling the uniform background data: **a** 11.5 kBq/ml (25% resampling), **b** 23 kBq/ml (50% resampling) and **c** 46 kBq/ml (100% resampling)

**Fig. 5:**
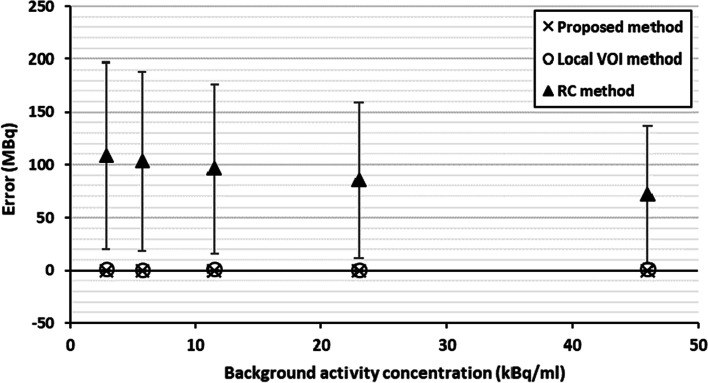
Absolute errors for the five sources to background ratios and associated uncertainties using the proposed method, RC method and local VOI method

Example images of the reconstructed composite data simulating different source to background images are shown in Fig. 4a–c. Figure 5 summarises the quantification results from these phantoms for the proposed, RC and local VOI methods.

The proposed method resulted in accurate quantification in a range of uniform background activity concentrations. The errors and uncertainties were consistent across the different uniform backgrounds. The largest errors were observed for the recovery coefficient approach. Uncertainties were also consistent for the RC method across the different uniform backgrounds as expected.

### Non-uniform background

**Fig. 6:**
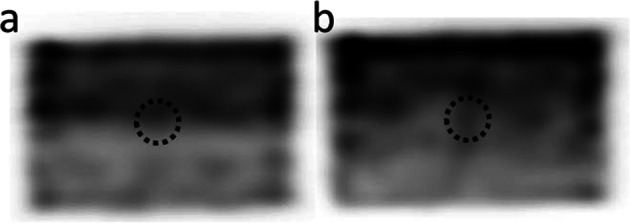
Reconstructed non-uniform background images generated using non-uniform Poisson resampling. Dashed circles indicate the approximate location of source of interest. **a** Down sampling in half of the phantom only, with smooth transition to no down sampling in second half of phantom (profile indicated by dashed line in Fig. 7). **b** Linear down sampling performed across the entire phantom length (phantom profile indicated by solid line in Fig. 7)

**Fig. 7:**
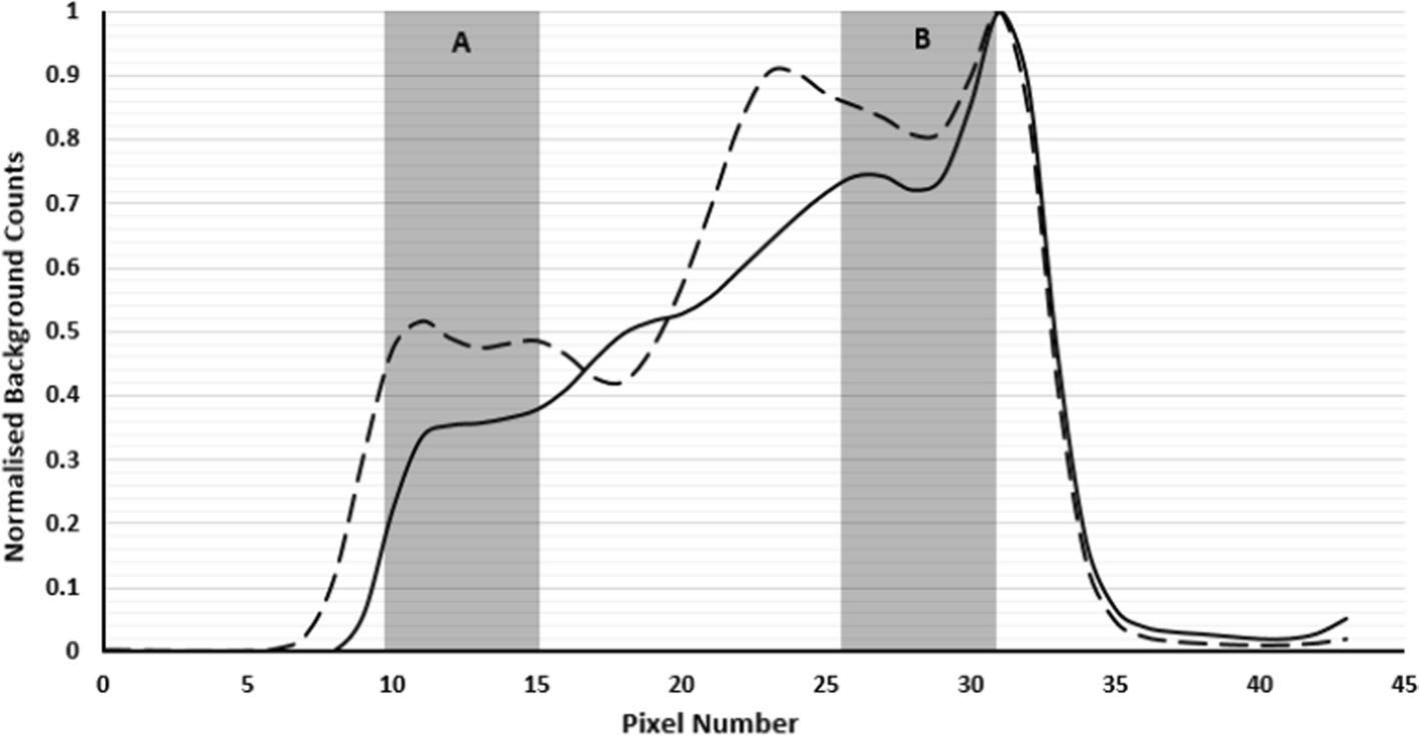
Vertical profiles of two of the reconstructed Poisson-resampled background data. Quantification using local VOI method was performed with background correction VOIs in two vertical locations indicated by the grey areas

**Fig. 8:**
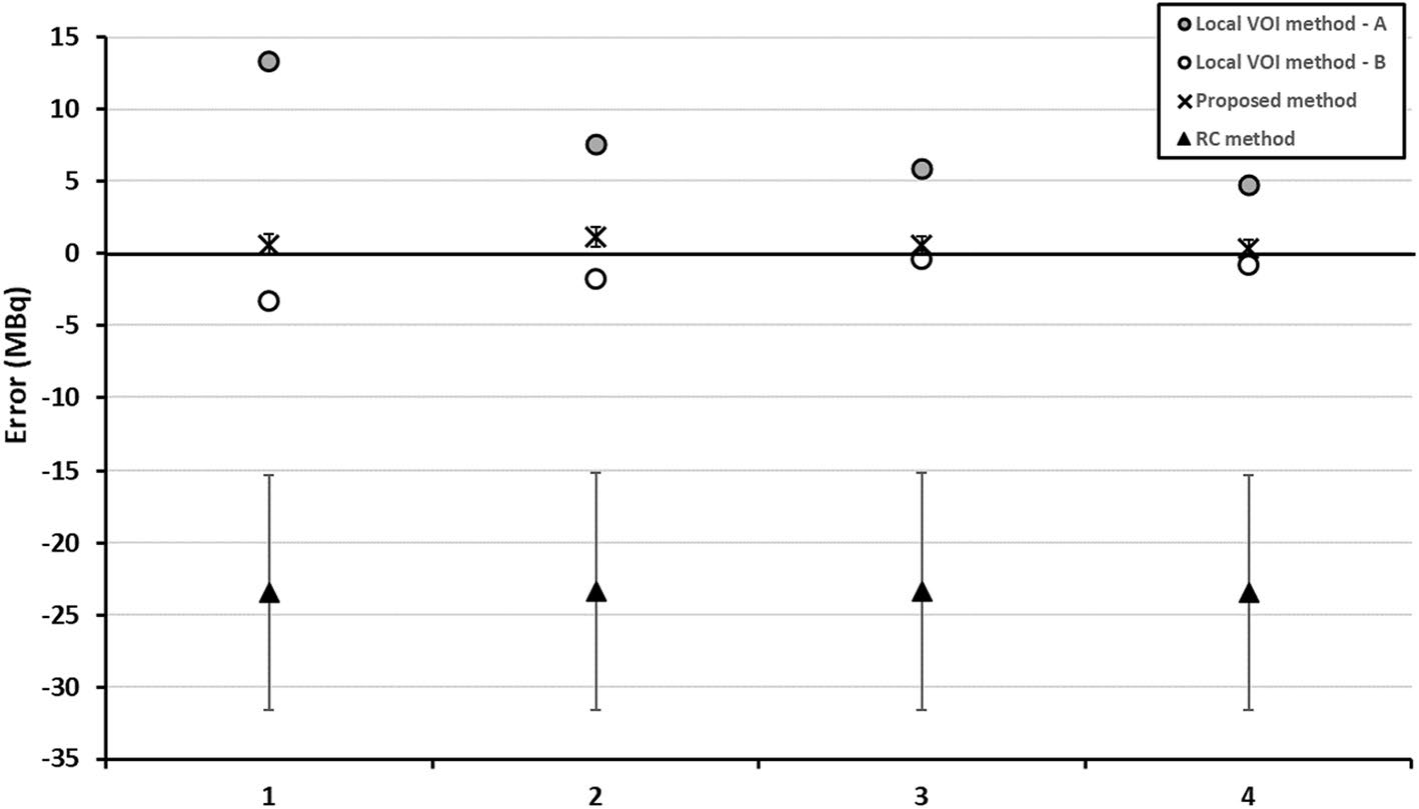
Absolute error and uncertainty in quantification of 33.3 MBq capsule using the proposed, RC method and local background VOI methods for 4 non-uniform background distributions. Local VOI method A and B used VOIs in different positions

The proposed method also resulted in accurate quantification of Na[^131^I]I sources in the presence of non-uniform background activity. Figure 6a, b shows reconstructed examples of the non-uniform background distributions generated by Poisson resampled data; Fig. 7 shows count profiles of the phantoms in vertical direction to illustrate the non-uniform background. Figure 8 summarises the quantification results of the 33.3 MBq capsule in four non-uniform backgrounds. For comparison, Fig. 8 also shows results using the local VOI and RC methods.

The proposed method resulted in accurate quantification within 1.2 MBq of the true source activity in the presence of a non-uniform background. The local VOI method was performed with the background correction VOI in two different phantom locations (A and B). The median percentage error in position A was 20.2% (range 14.2 – 39.9%) and − 4% (range − 1.9 to 9.9%) in position B. Errors in activity using the local VOI method were higher than the proposed method for all cases tested. Median errors and uncertainty results are summarised in Table 1 for all three methods (position A only for local VOI method). The recovery coefficient method was the least accurate of the three approaches. The errors and uncertainties were consistent across the different background distributions.

### Anthropomorphic phantom

**Fig. 9:**
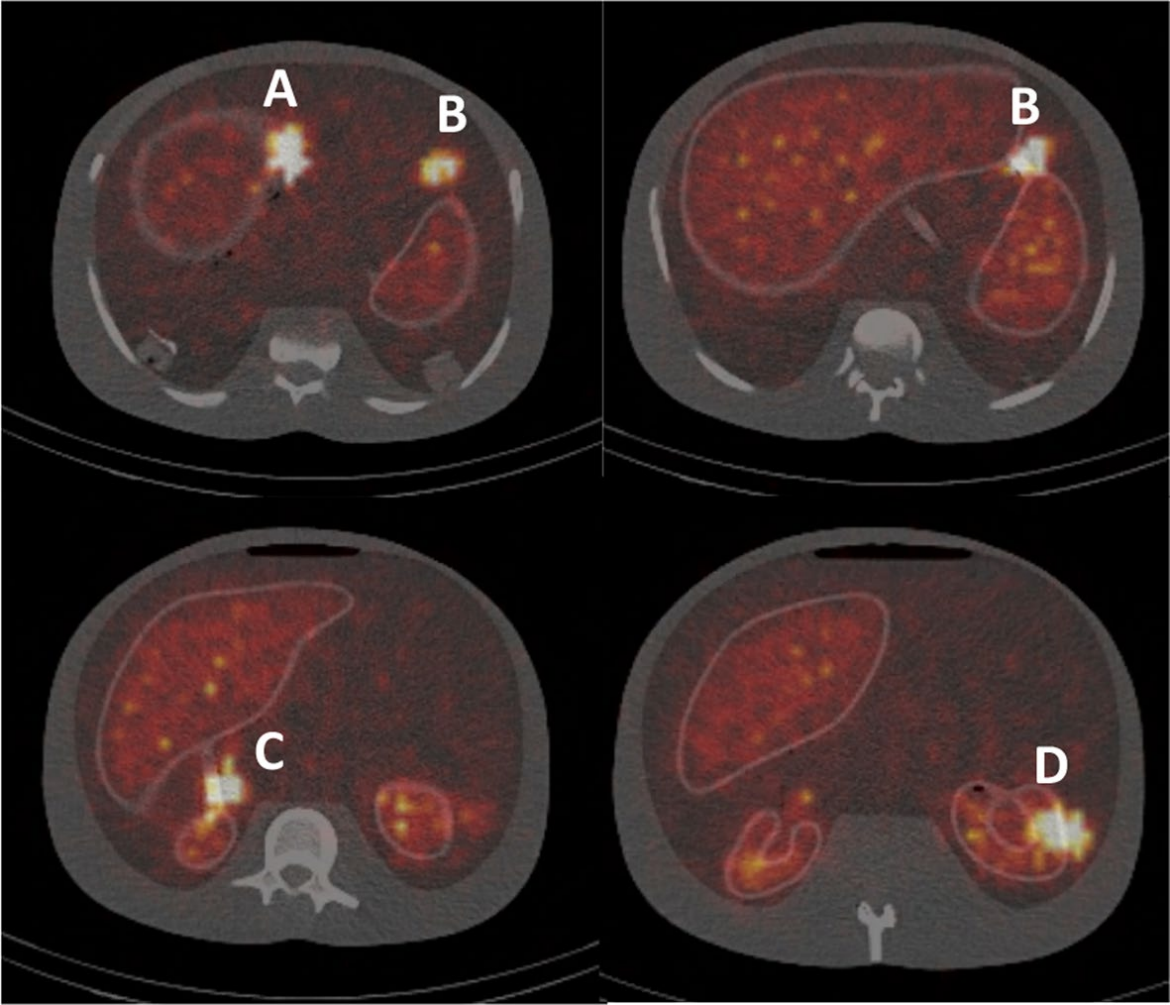
Transaxial images of the anthropomorphic phantom indicating the locations of the point sources

Figure 9 shows transaxial images of the phantom at each source location, labelled A to D. Results of source activity are presented in Table 2. The proposed methodology was able to quantify the activity in the lesion located at all locations to within 11%. The uncertainty estimate was generally representative of this error.

**Table 2:** Errors and uncertainties in quantification using the proposed method for four lesions in the anthropomorphic phantom

Lesion location	Error (MBq)	Error (%)	Uncertainty (%)
A	0.34	− 8.9	2.9
B	0.23	− 5.1	1.2
C	0.41	− 11.6	9.6
D	0.04	− 0.8	5.0

### Clinical case study

**Fig. 10:**
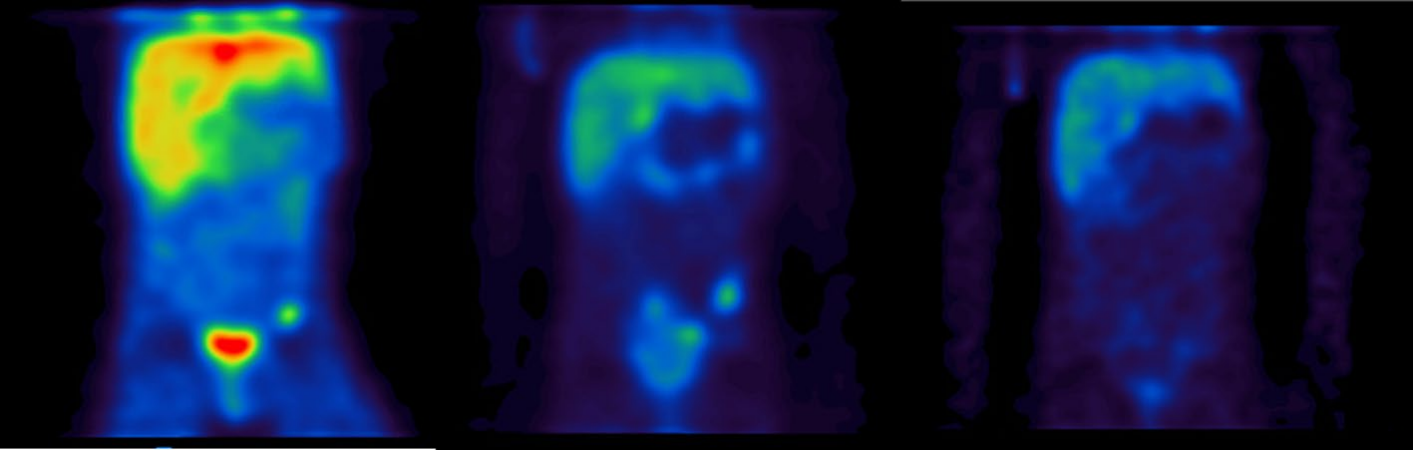
Coronal view of SPECT images at ~ 2 days, 5 days and 10 days. Quantification was performed on the bone metastases visualised in the left pelvic bone

**Fig. 11:**
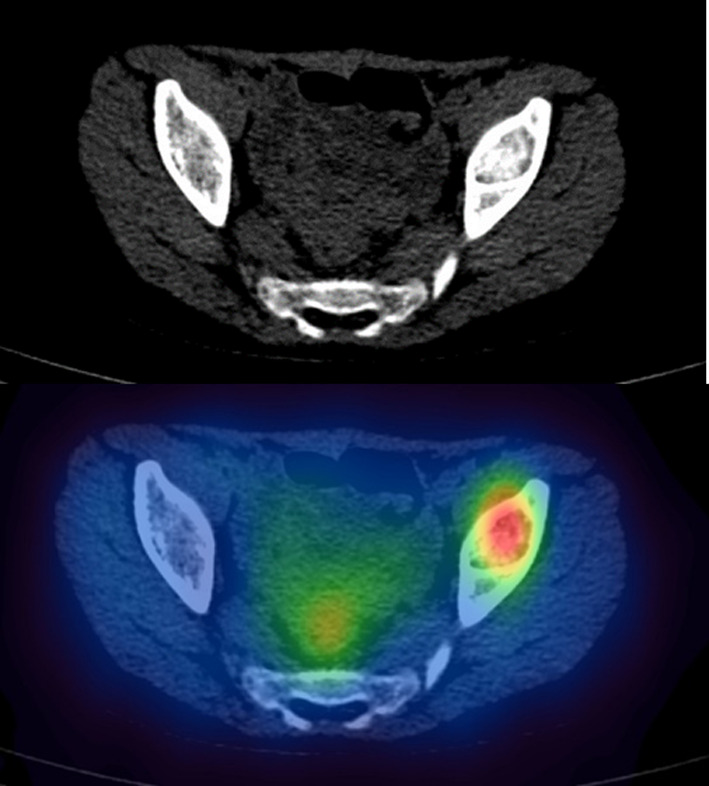
Axial CT and SPECT/CT of a bone metastases in [^131^I]mIBG patient

Example coronal images of the SPECT data at the three time points are shown in Fig. 10. A transaxial image with fused CT is shown in Fig. 11. The delineated VOI on the CT was 2.6 ± 0.3 cc. Uncertainty in volume was determined using the method described in the EANM guidance, for a CT voxel size of 0.73 × 0.73 × 0.8 mm. From Fig. 2, this volume requires a recovery coefficient of 0.31 ± 0.06.

**Fig. 12:**
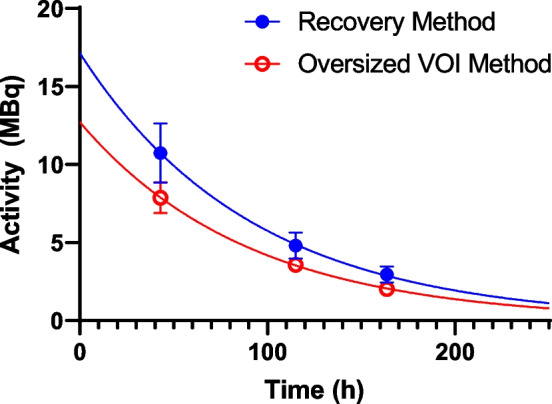
Time activity curve for bone metastases in [^131^I]mIBG radionuclide therapy patient

The calculated lesion activities with associated uncertainty at each SPECT time point are summarised in Table 3 for both quantification methods. Time activity curves for both data are given in Fig. 12. Effective half-life for both methods was identical, indicating a systematic difference between the methodologies. Time-integrated activities for each method were 1143 ± 13 and 1574 ± 26 MBq.hr for the proposed VOI and recovery methods, respectively. Calculated absorbed dose and uncertainty were 49.6 ± 5.5 Gy and 68.2 ± 13 Gy. Results for all dosimetric parameters fell within their uncertainty bounds for each method. The recovery method resulted in a higher estimate of activity and hence a higher estimate of absorbed dose in this case.

**Table 3:** Time, activity (*A*) and uncertainty in activity measurement *u*(*A*) for a bone metastases in an [^131^I]mIBG patient who underwent 3 × SPECT imaging after radionuclide therapy

Time (h)	Proposed method		Recovery method	
	*A*(*t*)	*u*(*A*)	*A*(*t*)	*u*(*A*)
43.0	7.88	0.10	10.74	1.91
115.1	3.58	0.05	4.80	0.84
163.7	2.02	0.05	2.96	0.52

## Discussion

The proposed alternative quantification and uncertainty methodology resulted in accurate quantification across a range of clinically relevant source distributions. Accurate ^131^I quantification was demonstrated without any background activity, in a range of uniform and non-uniform backgrounds and with lesions placed in an anthropomorphic phantom. Background physiological uptake is typical in clinical imaging, and a method for accurate correction is necessary to achieve accurate quantification. The uncertainty estimates for the clinical case presented here are comparable to those provided by Peters et al. [14] who performed small lesion dosimetry using an oversized VOI with a local VOI background correction.

Quantification with the recovery coefficient method resulted in inaccurate activity estimates for small volumes. The proposed methodology has shown superior quantification of small volumes when compared to other commonly used methodologies. Accurate activity quantification is an essential step in the dosimetry chain and might ultimately allow characterisation of dose–response relationships and avoid under- or overtreatment of patients [20].

Uncertainty estimates of activity quantification are important to provide confidence in the estimated absorbed doses especially when used in the context of personalised treatment planning. Peters et al. [14] and Finocchiaro et al. [17] have performed uncertainty analysis of lesion absorbed doses. The required accuracy will ultimately depend on the clinical question to be addressed, but the methodology proposed here provides the means to assess the uncertainty of absorbed dose estimates when considering sources that are smaller than the resolution of the SPECT system. Absolute uncertainties estimated for the method presented here appropriately represented the observed error and were significantly smaller than that of the common RC approach. This is likely due to the large uncertainties introduced when using recovery coefficients for small volumes.

Harmonisation of quantitative imaging methodologies including uncertainty calculations should lead to an increased ability for multi-centre collaboration [21] and meaningful investigations of dose–effect correlations [20] which have already been presented for several molecular radiotherapy treatments [22, 23]. The present work may aid in calculation of absorbed doses for small lesions, for which there is currently minimal guidance.

The local VOI and RC methods have several shortcomings when used for the quantification of small volumes as illustrated in Figs. 5 and 8. The accuracy of the RC method for small volumes largely depends on the chosen recovery function which is often generated with simplistic geometries such as spheres, and the accuracy of determining the metabolic active volume. The large deviations between the RC method and the true activities are likely due to inaccuracies arising from the RC fit at small volumes due to the lack of smaller phantom inserts and the difficulty in accurately assessing lesion size on SPECT/CT. This is reflected in the large uncertainty estimates for this methodology. These uncertainties underestimate the observed error, which can be explained by the fact that the linearisation of the problem using a first order partial derivative, as described in the EANM guidance, falls at large uncertainties and is no longer valid. The RC methods is also known to be less reliable when SPECT and CT are not aligned or if motion, such as respiratory or cardiac, affects the SPECT acquisition as the count distribution will be blurred, leading to an underestimation of total counts in the target volume.

Using a local background correction method [14] only works reliably in a perfectly homogenous background. Figure 7 shows that the position of the background VOI affects accuracy of the methodology in a non-uniform background. The proposed method’s use of concentric VOIs surrounding the source of interest should provide a better representation of the variation in local background and therefore improve this correction [13].

While the methodology has proven to work reliably in a series of scenarios presented here, the linear fit to the count data has potential limitations. The methodology is affected by the presence of hot sources in close proximity or in the presence of reconstruction artefacts, which can result in a nonlinear model. If more than three VOIs are drawn, it is possible to further extend this methodology and fit a nonlinear function to the data. This would then allow the user to more accurately model effects such as “spill-in” of counts from neighbouring background regions. The difficult here is that many more VOIs may be required, which unless automated would greatly increase processing time. In addition, the exact function to fit is undefined and would potentially have to change for every given scenario. We therefore chose to use a linear model and include these inaccuracies within the uncertainty estimation. In principle, the presented method is expected to work as well for larger volumes, but further work is required to expand the uncertainty schema presented here to these scenarios. The exact volume cut-off for which this approach should be used instead of the RC method, (or vice versa) has not been investigated in this study as it will likely depend on scanning and reconstruction parameters and vary for different radionuclides. Nevertheless, the presented method has proven to work well for quantification of small volumes, which is an area that has not previously been well studied and for which guidance is lacking.

## Conclusions

We have presented here a methodology for quantification of small target volumes in molecular radiotherapy including the associate uncertainty analysis. The method has been validated and proven to provide accurate results in a range of clinically relevant scenarios with varying background activity distributions.

## Supplementary Information

**Additional file 1.** Schema for propagation of uncertainty in activity to that associated with absorbed dose.

## Data Availability

Data can be provided upon a reasonable request to the corresponding author.

## Abbreviations

SPECT: Single-photon emission computed tomography
PVE: Partial volume effect
MRT: Molecular radiotherapy
VOI: Volume of interest
CT: Computed tomography
EANM: European Association of Nuclear Medicine
LPU: Law of propagation of uncertainty
RC: Recovery coefficient
HEGP: High-energy general purpose
