# Supplementary material for: Quantification and dosimetry of small volumes including associated uncertainty estimation
Source: EJNMMI Phys. 2022 Dec 13;9:86. doi: 10.1186/s40658-022-00512-9 (PMC9748012; doi:10.1186/s40658-022-00512-9)
Supplement: Supplementary file 1 — Additional file 1. Schema for propagation of uncertainty in activity to that associated with absorbed dose. [file 40658_2022_512_MOESM1_ESM.docx]

# Supplementary Material: Quantification and dosimetry of small volumes including associated uncertainty estimation.

Lily Carnegie-Peake, Jan Taprogge, Iain Murray, Glenn D. Flux and Jonathan Gear

The following section describes the propagation of uncertainty in activity to that associated with absorbed dose. As the methodology to determine activity is different to that presented in the EANM guidance document, there are slight differences in the resulting uncertainty estimates which ultimately leads to a simpler expression of absorbed dose uncertainty.

When fitting a time- activity curve to determine time-integrated-activity, $\tilde{A}$, components of uncertainty can be categorised due to random or systematic effects. Measurements of counts are independent of each other and should be treated as random effects. However, due to the complexity of the many other non-modelled random effects, it is the causality of these imperfections is measured and derived from the uncertainties in the fit parameters of the TAC. Here, non-linear regression techniques are used to minimize the objective function

| $\chi^{2}=\sum\left[ \boldsymbol{E} \right]^{2}=\sum\left[ A_{i}-f(t_{i}) \right]^{2},$ | (1) |
| --- | --- |

A weighting term, to account for count uncertainty, can included in the iterative minimisation algorithm, such as a weighted Gauss-Newton algorithm

| ${\boldsymbol{p}^{i+1}=\left[ \boldsymbol{J}_{\boldsymbol{p}}^{\boldsymbol{\top}}\boldsymbol{V}_{\boldsymbol{C}}\boldsymbol{J}_{\boldsymbol{p}} \right]}^{-1}\left[ \boldsymbol{J}_{\boldsymbol{p}}^{\boldsymbol{\top}}\boldsymbol{J}_{\boldsymbol{p}}\boldsymbol{E} \right]+\boldsymbol{p}^{i}$ | (2) |
| --- | --- |

where

| $\boldsymbol{V}_{\boldsymbol{C}}\mathbf{=}\left[ \begin{matrix} u^{2}(C_{1}) & \ldots& 0 \\ \vdots& \ddots& \vdots\\ 0 & \ldots& u^{2}(C_{n}) \end{matrix} \right]$ | (3) |
| --- | --- |

is the input covariance matrix, associated with count uncertainty, $\boldsymbol{p}$ is the matrix of TAC parameter estimates, and $\boldsymbol{J}_{\boldsymbol{p}}$ is the matrix of first‑order partial derivatives of the TAC model with respect to $\boldsymbol{p}$, evaluated at each $A_{i}$.

The covariance matrix $\boldsymbol{V}_{\boldsymbol{p}}$ for the estimates of the TAC parameters is equal to that given in the EANM guidance [16]

| $\boldsymbol{V}_{\boldsymbol{p}}=\frac{\chi^{2}}{n-q}\left[ \boldsymbol{J}_{\boldsymbol{p}}^{\boldsymbol{\top}}\boldsymbol{J}_{\boldsymbol{p}} \right]^{-1}$ | (4) |
| --- | --- |

Which can be used to determine the random component of uncertainty in $\tilde{A}$

| $u_{r}^{2}\left( \tilde{A} \right)=\boldsymbol{g}_{\boldsymbol{p}}^{\top}\boldsymbol{V}_{\boldsymbol{p}}\boldsymbol{g}_{\boldsymbol{p}}$ | (5) |
| --- | --- |

For a single exponential function described by $\boldsymbol{=}\left[ A_{0},\lambda\right]^{\top}$ .

| $\boldsymbol{g}_{\boldsymbol{p}}^{\top}=\left[ \begin{matrix} \frac{\partial\tilde{A}}{\partial A_{0}}, & \frac{\partial\tilde{A}}{\partial\lambda} \end{matrix} \right]=\left[ \begin{matrix} \frac{1}{\lambda}, & -\frac{A_{0}}{\lambda^{2}} \end{matrix} \right].$ | (6) |
| --- | --- |

Hence

| $\left[ \frac{u_{r}(\tilde{A})}{\tilde{A}} \right]^{2}=\left[ \frac{u(A_{0})}{A_{0}} \right]^{2}{+\left[ \frac{u(\lambda)}{\lambda} \right]}^{2}-2\frac{u\left( A_{0},\lambda\right)}{A_{0}\lambda}$ | (7) |
| --- | --- |

The combined uncertainty associated with random and systematic effects is then expressed as,

| $u^{2}\left( \tilde{A} \right)= u_{r}^{2}\left( \tilde{A} \right)+u_{s}^{2}\left( \tilde{A} \right)$ | (8) |
| --- | --- |

Such that,

| $\left[ \frac{u(\tilde{A})}{\tilde{A}} \right]^{2}=\left[ \frac{u(A_{0})}{A_{0}} \right]^{2}{+\left[ \frac{u(\lambda)}{\lambda} \right]}^{2}-2\frac{u\left( A_{0},\lambda\right)}{A_{0}\lambda}+\left[ \frac{u\left( Q \right)}{Q} \right]^{2}$ | (9) |
| --- | --- |

Note that the uncertainty in the measured counts is not included in expression (9) as it is already accounted for in the estimated uncertainty of the fit parameters. In addition none of the terms in expression () are dependent on the physical volume of the lesion or organ. Therefore, unlike the situation described in the EANM guidance, time-integrated-activity and the S-value remain mutually independent. The standard uncertainty in absorbed dose is simply the respective fractional standard uncertainties added in quadrature.

| $\left[ \frac{u(\bar{D})}{\bar{D}} \right]^{2}=\left[ \frac{u(\tilde{A})}{\tilde{A}} \right]^{2}{+\left[ \frac{u(S)}{S} \right]}^{2}=\left[ \frac{u(\tilde{A})}{\tilde{A}} \right]^{2}{+\left[ \frac{u(m)}{m} \right]}^{2}$ | (10) |
| --- | --- |

Where $S$ is the S-value and methods to derive values for $u(S)$ are described elsewhere.
